# Effects of Neuromuscular Training Applied During Ramadan on Physical Fitness and Injury Prevention in Highly-Trained Male Youth Soccer Players

**DOI:** 10.1186/s40798-025-00831-y

**Published:** 2025-04-07

**Authors:** Ali Belamjahad, Claire Tourny, Anthony C. Hackney, Fatiha Laziri, Ayoub Saeidi, Ouafae El Hachimi, Ismail Laher, Urs Granacher, Hassane Zouhal

**Affiliations:** 1 1CETAPS UR 3832 (Research Center of Sport Science), University of Rouen Normandy, Rouen, France; 2 Department of Exercise and Sport Science, Department of Nutrition, University of North Carolina, Chapel Hill, NC USA; 3 Laboratoire Ecologie, Environnement et Santé Equipe Santé Humaine et Environnement Faculté des Sciences de Université Moulay Ismail, Meknes, Morocco; 4 Department of Physical Education and Sport Sciences, Faculty of Humanities and Social Sciences, University of Kurdistan, Sanandaj, Kurdistan Iran; 5 Higher Institute of Nursing Professions and Health Techniques, Oujda, Morocco; 6 Department of Anesthesiology, Pharmacology and Therapeutics, The University of British Columbia, Vancouver, Canada; 7 Department of Sport and Sport Science, Exercise and Human Movement Science, University of Freiburg, Freiburg, Germany; 8 M2S (Laboratoire Mouvement, Sport, Santé), Université Rennes, Rennes, France; 9 Institut International des Sciences du Sport (2I2S), 35850 Irodouer, France

**Keywords:** Football, Muscular fitness, Intermittent fasting, Injuries

## Abstract

**Background:**

The intermittent fasting period of Ramadan is associated with reductions in training volumes and intensities to maintain physical fitness levels and reduce injury occurrence. Accordingly, it might be beneficial to include neuromuscular training (NMT) applied during Ramadan to avoid detraining and promote injury prevention in soccer players. This study aimed to analyze NMT effects on physical fitness and injury prevention during the Ramadan fasting period in young soccer players.

**Methods:**

Forty young highly-trained male soccer players (U17/U19) were randomly assigned to a NMT (n = 20) or a traditional soccer-specific training group (TT, n = 20). NMT was conducted during Ramadan, lasted four weeks, and included two weekly sessions with exercises to promote muscle strength, power (plyometrics), speed, balance. TT contents were similar to the training period before Ramadan and comprised two weekly sessions including endurance and sprint-based exercises. The training volume was similar between groups. Body composition (body fat), linear sprint (5-m, 10-m, 30-m sprint), and change-of-direction (CoD) speed (T-test with and without ball), muscle power (squat [SJ], countermovement jump [CMJ]), peak isokinetic torque of the knee flexors, extensors, and soccer-specific performance (Loughborough soccer passing test [LSPT], Yoyo intermittent test level 1 [YYIRT L1], repeated-shuttle-sprint ability test [RSSA]) were determined before and after the Ramadan period. The overall injury rate per 1000 h of exposure (training, match) was carried out during and after the four-week Ramadan period and until the end of the soccer season (overall 8 weeks).

**Results:**

No significant between group baseline differences were noted. Group-by-time interactions were significant for most assessed variables (0.001 < *p* < 0.004, 0.22 < d < 0.76) in favor of NMT. Variations in body mass, body fat, and BMI determined by post-hoc tests indicated significant decreases in NMT but not TT (0.026 < *p* < 0.047, 0.65 < d < 0.73). Moreover, post-hoc tests showed that NMT provided linear and COD speed improvements, enhanced muscle power (SJ, CMJ) and improved soccer-specific performance (*p* < 0.001, 0.71 < d < 2.53). Additionally, post-hoc tests revealed significant isokinetic strength increases in favor of NMT for all peak torque variables (0.015 < *p* < 0.049, 0.64 < d < 0.81). The overall injury rate was significantly lower in NMT (8.00/1000 h exposure) compared to TT (13.33/1000 h exposure) (*p* = 0.049; d = 0.66).

**Conclusions:**

Findings suggest that a four-week NMT conducted during Ramadan fasting helped to maintain or even improve measures of physical fitness including isokinetic strength. In addition, significant NMT-related reductions in injury occurrence were noted in highly-trained young male soccer players.

## Key points

- Our results indicate that the integration of 45 to 60 minutes of neuromuscular training, two times per week, during the month of Ramadan fasting, can improve physical fitness in highly-trained young male soccer players.
- This study demonstrated that neuromuscular training has the potential to reduce the incidence injury rate and the burden compared to traditional soccer-specific training during Ramadan.
- Based on the study outcomes, neuromuscular training appears to be an effective training approach during the month of Ramadan fasting to improve selected physical fitness parameters and to reduce injury occurrence in highly-trained male soccer players.

## Background

Ramadan is the ninth month of the Islamic lunar calendar, during which healthy pubescent and older Muslims voluntarily abstain from consuming food or liquids from sunrise until sunset, although there are no such restrictions at night [1]. A day of fasting can vary from 12 to 18 h depending on the geographic location and the season in which Ramadan takes place, with fasting days being the longest (lasting up to 18 h) in the summer months, shorter periods of fasting (~ 12 h daily) occurring during the winter months [2]. The rhythm of daily life during Ramadan is disrupted by the practice of fasting during the day and spiritual and social activities during the evening [3]. Sleep quality accompanied by a reduction in sleep time is frequent during the fasting period [4], due to the delay of evening meals and the eating of breakfast (Suhur) very early before dawn [5]. This disorganization of the sleep cycle can induce a drop in physical performance that can continue beyond two weeks after Ramadan [6]. Additionally, Ramadan intermittent fasting may negatively affect the players' physiology, biochemistry, and behavior [7]. However, findings on the effects of Ramadan intermittent fasting on physical performance are contradictory [8], with some reporting no changes [9], while others documenting decreases in physical performance [10].

Soccer is an intermittent activity that requires players to cover long distances (10–13 km per match) and undertake regular high-intensity efforts (approximately 1300 individual activities lasting 4–6 s each) over 90 min matches [11]. Players must be able to perform explosive and randomly repeated actions, such as linear sprints, quick changes-of-directions (CoD) including decelerations and accelerations, and jump actions during the different phases of match play [12]. These soccer-specific performance determinants can be improved through neuromuscular training (NMT) which is a multimodal training modality including exercises to promote muscle strength and power, linear- and CoD speed, balance [13, 14]. There is ample evidence from original research and systematic reviews with meta-analyses that NMT has the potential to improve physical fitness and reduce injury occurrence in young athletes [15, 16].

The need to consider specific training modalities such as NMT during the period of Ramadan is that most sport competition schedules do not take religious rituals into account. Accordingly, Ramadan can coincide with competitions, as was the case with the London Olympic Games (2012), the FIFA 2014 and 2018 World Cups, the 2018 UEFA U19 championships, and the 2018 final of the UEFA Champions League. Accordingly, Muslim soccer players need to find strategies to cope with the demands of match play and training during the Ramadan period. To enable Muslim players to compete and train during Ramadan, coaches deliberately reduce training loads and intensities to maintain physical fitness and avoid injuries [17].

The few studies that have examined the effects of strength training combined with intermittent fasting have primarily focused on the timing or volume of strength training [18–20]. To our knowledge, no studies are available that explored the impact of different strength and conditioning programs during the intermittent fasting period. Thus, Ramadan-specific programming strategies may contribute to minimize the negative impact of reduced training loads and intensities. Here, we aimed to examine the effects of NMT versus traditional soccer-specific athletic training (TT) on selected measures of physical fitness and injury occurrence in highly-trained male youth soccer players. We hypothesized that NMT would be more effective than traditional training modalities in enhancing physical fitness and reducing injury rates when applied during the Ramadan period in young male soccer players [13–16].

## Methods

### Participants

Forty young male soccer players from U17 (n = 20) and U19 (n = 20) teams were recruited from a professional soccer club to participate in this study. According to McKay et al. [21], the players are categorized as Tier 3 (highly-trained, national level). Goalkeepers were excluded from the study. Players were 16.0 ± 0.6 years (U17) and 17.6 ± 0.7 years (U19) old. In accordance with the guidelines of Mirwald et al. [22], players were categorized as post-peak-height-velocity (PHV) with U17: + 1.7 ± 0.7 years and U19: + 2.9 ± 0.9 years (Table 1). Maturity offset was classified according to the procedures defined by Sherar et al. [23]. Chronological age was calculated by subtracting the player's date of birth from the current date. This group consists of players with an average training volume of 7–10 h per week distributed over 5–8 weekly sessions, supplemented by a weekly match. Training contents varied according to season objectives and competition periods. The season extended over 10 months, from September to June, with a 15-day winter break.

**Table 1:** Characteristics of the study sample

Group	Number	Age at PHV (years)	Maturity offset (years)	Chronological age (years)	Soccer practice (years)
TT (n = 20)	U17 (n = 10)	14.6 ± 0.93	1.6 ± 0.87	16.2 ± 0.51	2.2 ± 0.89
	U19 (n = 10)	14.7 ± 0.56	3.0 ± 0.70	17.7 ± 0.49	3.5 ± 0.41
NMT (n = 20)	U17 (n = 10)	14.1 ± 0.49	1.8 ± 0.61	16.0 ± 0.62	2.1 ± 0.69
	U19 (n = 10)	14.8 ± 0.42	2.7 ± 1.02	17.5 ± 0.95	3.7 ± 0.32

*TT* traditional training, *NMT* neuromuscular training, *PHV* peak height velocity [22]; Values = mean ± SD

**Fig. 1:**
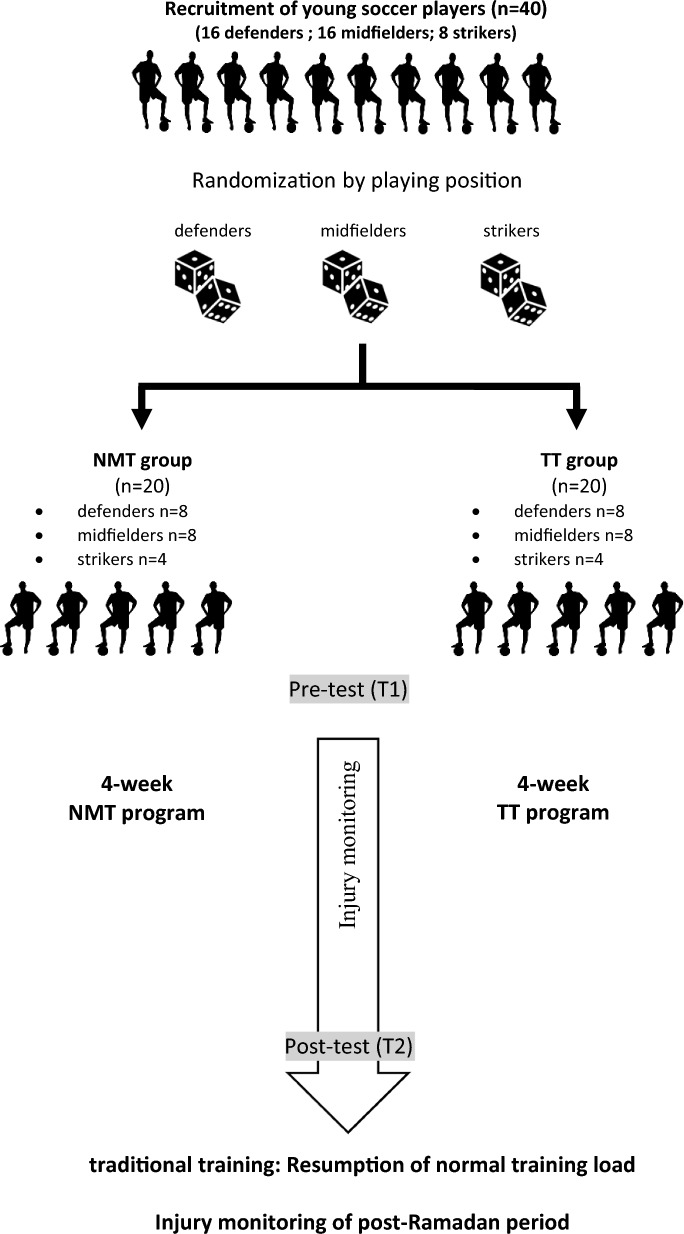
Flow chart of the study design. *TT* traditional training; *NMT* neuromuscular training

Players were randomly assigned to NMT or TT based on their playing positions (16 defenders, 16 midfielders, and 8 attackers) using randomization software (Randomization.com). All researchers responsible for testing were blinded to group allocation (Fig. 1).

TT and NMT were conducted during Ramadan (April–May 2023) and lasted four weeks with two weekly NMT and TT sessions. In addition to NMT and TT, players participated in technical/tactical soccer training sessions. During the intervention period, players performed five weekly sessions, including two sessions of the NMT/TT program and one friendly game per week. Players were informed of the study protocol, potential risks, and benefits before signing the consent form. The study was developed following the ethical standards of the Declaration of Helsinki and was approved by the Local Ethics Committee of the club (Renaissance sportive de Berkane), Morocco.

A sample size of 38 players was estimated using an a priori power analysis (G ∗ Power, Version 3.1, University of Düsseldorf, Germany). The power analysis was calculated for the primary outcome (i.e., squat jump height) with an assumed power of 0.90, an alpha level of 0.01, and a medium effect size of Cohen’s *f* = 0.33 based on a related study [24]. Therefore, we recruited additional players (N = 40) to allow for potential drop-outs.

### Dietary Intake Program

All participants lived in the youth soccer academy (Berkane, Morocco) and received a daily diet defined by the nutritional staff to ensure adequate fluid and nutrient intake (Table 2). The dietary intake program was quantified using the Moroccan Food Composition Tables (2019).

**Table 2:** Dietary intake program for both experimental groups during the Ramadan intermittent fasting period (means ± standard deviations [SDs])

	Dietary intake
Carbohydrate (g/day)	289.5 ± 61.6
Carbohydrate (%)	50.3 ± 5.3
Protein (g/day)	98.6 ± 20.9
Protein (%)	19.7 ± 2.7
Fat (g/day)	62.4± 20.9
Fat (%)	26.8 ± 4.6
Total water intake (L/day)	4.2 ± 1.6
Energy intake (kcal/day)	2407.3 ± 643.8

### Study Design

**Fig. 2:**
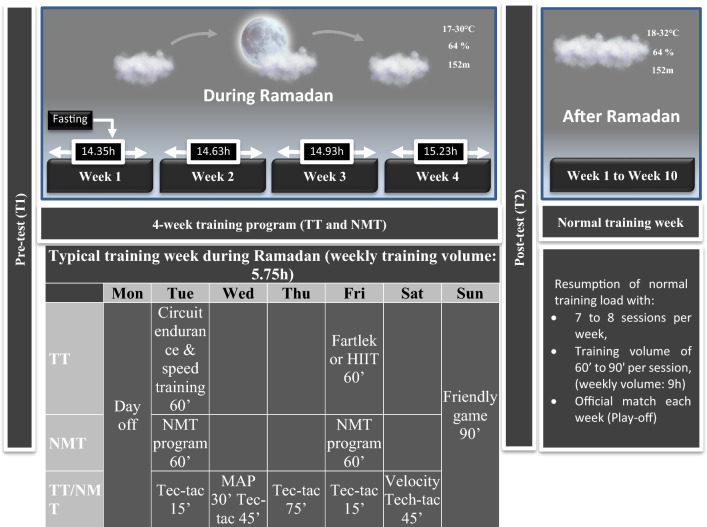
Flow chart of the training period, test organization and injury monitoring. *TT* traditional training; *NMT* neuromuscular training; *HIIT* high intensity interval training; *Tec-tac* technical-tactical; *MAP* maximum aerobic power

Players were tested for anthropometrics, body composition, and physical fitness before (T1) and after (T2) the four-week training period. Two recovery days were allowed after the pre-test (T1) and before the post-test (T2). The average duration of the daily fast during Ramadan was approximately 14.8 h. The study was conducted in Morocco (Berkane), where daytime temperatures ranged from 17–30 °C and relative humidity was ~64% (Fig. 2).

### Body Composition

Body height was measured in centimeters (cm) using a stadiometer with an accuracy of 1 mm (SECA 206 ®), body mass in kilograms (kg) using an electronic scale with an accuracy of 0.1 kg (KINLEE ®), and body fat percentage using a Lunar iDXA instrument (GE Medical Systems, Madison, Wisconsin, USA). The body mass index (BMI) was calculated as body mass (kg) divided by height in meters squared (kg/m^2^).

### Measures of Physical Fitness

Physical fitness was tested before (T1) and after (T2) the four weeks NMT or TT using field tests and an isokinetic dynamometer (Fig. 2). On the first test day, squat (SJ) and countermovement jump (CMJ) tests as well as the T-test with and without ball and the Yoyo intermittent run test level 1 (YYIRT L1) were performed. On the second test day, linear sprint tests (5-, 10-, 30-m), the Loughborough soccer passing test (LSPT), and the repeated-shuttle-sprint ability test (RSSA) were conducted. On the third test day, anthropometric, body composition, and isokinetic tests were performed. All participants were familiar with the testing protocol.

Before testing, a standardized 10-min warm-up consisting of submaximal running, jumping, and active-dynamic stretching exercises was performed. The field tests took place on a third-generation synthetic pitch. All tests were monitored by the same examiner at T1 (pre-test) and T2 (post-test). The test–retest values were calculated from the data collected during pre and post intervention.

#### Muscle Power Tests

Participants performed SJ and CMJ tests to measure proxies of lower limb muscle power. For the SJ tests, players had to remain in a static position for 1 s with a 90° knee flexion angle before executing a maximal vertical jump. For the CMJ tests, players started from an upright erect standing position and had to perform a downward movement to a semi-squatted position followed by a full extension with maximal vertical acceleration. For each jump test, players performed two trials separated by an interval of 60 s, and the hands were placed at the supra-iliac zone during the jumps. The best of two trials was used for further analysis. Jump height was assessed using a portable force platform (Fusion Smart Speed Jump, Fusion Sport, Australia), and participants always performed their jumps in the same order (SJ followed by CMJ). The intra-class correlation coefficients (ICC) for test–retest reliability were 0.936 (0.832–0.973) for SJ and 0.958 (0.926–0.976) for CMJ.

#### Isokinetic Strength Tests

Peak torque of the knee extensors and flexors was tested using an isokinetic dynamometer (Con-Trex®). The test was preceded by a 5 min standardized warm-up consisting of unloaded knee flexion–extension exercises on the isokinetic dynamometer. All tests were carried out by the same examiner at T1 and T2. The players were in a seated position on the dynamometer and fixed with three belts with the arms in neutral position alongside the body. During testing, a hip angle of 100° was used relative to the trunk. The tested leg was fixed on the seat of the dynamometer using a strap and the knee axis was adjusted so that it matched the axis of rotation of the dynamometer [25]. The test was carried out at three angular velocities (30°/s, 60°/s, 240°/s). We evaluated the concentric peak torque of the knee flexors (hamstring) and extensors (quadriceps) at 60°/s and 240°/s and the eccentric peak torque at 30°/s. For each test, the players had to perform three maximal trials at angular velocities of 60°/s and 30°/s and five trials at 240°/s. The best trial was taken for further analysis. The same procedure was performed for the dominant and non-dominant leg. The dominant leg was determined through a ball kicking test. The results were expressed in absolute values (Nm). We calculated the conventional and functional ratios to assess unilateral lower limb imbalances. The conventional Hcon(240°/s)/Qcon(240°/s) and functional Hecc (30°/s)/Qcon (240°/s) ratios were computed in accordance with Baroni et al. [26]. To determine pathological thresholds and identify injury risks, we used the procedure described by Croisier et al. [27]. For conventional ratios, cut-offs must be > 0.45 and for the functional ratio cut-offs must be > 0.89. The ICCs for test–retest trials for all indices of maximal torque (30, 60, 240°/s) were between 0.848 and 0.862.

#### Linear Sprint Speed Tests

We evaluated 5-m, 10-m, and 30-m linear sprints from a standing start position. Players were instructed to perform maximal sprints over a distance of 30-m and recorded split times of 5-m and 10-m with a 60 s rest between trials. The split times were recorded using four photocell gates (Smart Speed, Fusion Sport, Australia) placed at three distances from the start line. The fastest time for each distance was used for further analysis. The ICC for test–retest reliability for 5-m, 10-m and 30-m performances was 0.842, 0.895 and 0.914, respectively.

#### Change-of-Direction Speed Test

We measured the players' ability to quickly change directions using the T-test [28]. The test is composed of several movements with CoD to the right, left, and backward in a “T” shape. Players started with both feet behind the starting line. The test was timed using a photocell system (Smart Speed, Fusion Sport, Australia). Each player performed two sprints with 60 s recovery between trials. Players completed two trials and the shortest time was used for analysis. The ICC for test–retest trial for CoD sprint with the ball and sprint without the ball, was 0.843 and 0.896, respectively.

#### Soccer-Specific Performance Tests

##### Yo–Yo Intermittent Recovery Level 1 (YYIRT L1)

This test evaluates players’ aerobic and recovery capacities during high-intensity intermittent efforts. The test was carried out on an outdoor synthetic pitch. The players had to complete runs of 2 × 20 m (round trip) at increasing speeds with a 10-s recovery period between runs. The pace was facilitated by an audible signal. The speed increased in stages, making the test more and more demanding as it progressed. The test stopped when the player could no longer reach the line before the sound signal on two consecutive occasions or until exhaustion. The player had to complete a 10-min warm-up before starting the test. The distance covered (m) was recorded as the test result [29]. The ICC for test–retest reliability was 0.984 for the distance covered.

##### Repeated Shuttle Sprint Ability (RSSA)

We performed the repeated shuttle sprint ability test (RSSA) to assess players’ capacity to repeat high-intensity intermittent efforts. This test involves performing 6 sprints over 40-m distances with a CoD (20-m outward and 20-m return) at maximum speed interspersed with a 20-s recovery. Sprint time was recorded using a photocell system (Smart Speed, Fusion Sport, Australia). The shortest time was used for further analysis. The percentage decrease in performance (RSSA decrease) was calculated according to the equation: RSSA decrease = ([average RSSA]/[best RSSA] × 100)–100) [30]. The ICC for test–retest reliability was 0.912 for RSSA best, and 0.931 for RSSA decrement.

##### Loughborough Soccer Passing Test (LSPT)

This test was designed to assess the specific technical quality of soccer players according to the protocol described by Ali et al. [31]. The players had to make a series of sixteen passes against four benches arranged in a rectangle, and a colored zone (red, blue, green, and white) of 60 × 30 cm fixed in the center of each bench, serving as a target zone. The players had to make random passes against the bench of the color announced by a coach. Players had to complete the sixteen passes as quickly as possible with as few errors as possible. The series of passes was determined by one of eight trial commands randomly generated by the trainer, such that each series of passes consisted of eight short passes (3.5 m; white and red) and eight long passes (4 m; green and blue). LSPT performance includes the result of the time taken to complete all 16 passes (original time), time added for errors and inaccurate passes (penalty time), and execution time (original time + time penalty). The penalty time was estimated according to the type of error; i.e., missed bench or pass to the wrong color (+ 5 s)/pass outside the target zone or manipulated by hand (+ 3 s)/pass made outside the target zone outside the central passing zone or touches a cone (+ 2 s)/for each second which exceeds the authorized time of 43 s (+ 1)/a bonus for each precise pass which touches the central strip of 10 cm cited in the middle of the target area (− 1). Time was measured using a manual stopwatch. Players completed two trials and the best time was used as the performance score. The ICC for test–retest reliability for total time, penalty time, and performance time was 0.843, 9.896, and 0.921, respectively.

### Hooper Questionnaire

The well-being of the players during the period of Ramadan intermittent fasting were monitored using the Hooper’s Index [32]. For this purpose, alterations in daily rhythms of sleep quality and general fatigue were recorded. Players were asked to complete a self-assessment questionnaire on a 1–7 scale reporting perceived sleep quality, perceived quantities of stress, delayed-onset of muscle soreness (DOMS), and general fatigue at the same time of day, i.e. morning. The rating scale ranged from “very, very good” to “very, very bad” for sleep, and from “very, very low” to “very, very high” for DOMS, stress, and fatigue. The sum of the four answers was used to calculate the Hooper’s Index.

### Training Load

#### Internal Training Load: Ratings of Perceived Exertion (RPE)

The assessment of the internal training load was determined using a modified version of Borg’s rating of perceived exertion (session-RPE, sRPE) with a scale of 0–10 for the entire training session. The players were asked to provide their feelings within 15–20 min after the end of each training session. The sRPE was determined by multiplying the RPE for the training session by the duration of the session. The load equation  = sRPE × time (expressed in arbitrary units; A.U.) [33].

#### Monitoring of External Training Load Using a Global Positioning System (GPS)

In accordance with Zouhal et al. [12], the estimation of external training load was quantified using GPS data (Gpexe®. Italy) on the distance covered at different speed intensity levels. The GPS device provides position, velocity, and distance data collected at a sampling frequency of 15-Hz. Each player wore the device inside a custom-made vest supplied by the manufacturer across the upper back between the left and right scapulae. All devices were activated 30 min before data collection to allow acquisition of satellite signals as per manufacturer’s instructions. After each training session or game, GPS data were downloaded, using GPEXE software, on a personal computer and further analyzed. Data recorded before the start of the season indicated that the devices have high inter-unit variability. Differences (± 90% CI) between the units ranged from 8.7 ± 3.5 to 10.8 ± 13.5% for straight line running movements and from 9.3 ± 6.9 to 11.5 ± 6.8% in the CoD courses.Therefore, each player wore the same GPS device for each training session and game to avoid this variability. We adopted the speed thresholds used by FIFA during the Qatar 2022 World Cup: Zone 1 (0–6 km/h), Zone 2 (6–15 km/h), Zone 3 (15–20 km/h), Zone 4 (20–25 km/h) and Zone 5 (> 25 km/h). The recorded distances were reported in absolute values (meters). The number of sprints (> 25 km/h), accelerations (+ 3 m/s^2^) and decelerations (− 3 m/s^2^) were gathered. On average, the number of satellites connected during data recodring was 11, and the average horizontal dilution of precision (HDOP) was 0.8.

### Injury Occurrence

Injuries occurring during and after the Ramadan period until the end of the soccer season (overall eight-week monitoring period) were recorded and classified per the Fédération Internationale de Football Association (FIFA) consensus statement. Injuries were collected in an Excel database, containing the type (fractures and bone stress/joint and ligament/joints/muscle and tendon/contusions/laceration and skin lesions/central, peripheral nervous system/other injuries), location (head and neck/ upper limbs/ trunk/ lower limbs). Injury severity has been defined based on the number of days a player could not participate in training: minimal injury corresponds to 1–3 days of absence, slight injury to 4–7 days, moderate injury to 8–28 days, and serious injury to more than 28 days [34].

### Training Programs

Players completed five weekly training sessions and one friendly match every week during the four-week period of the study. The training sessions took place in the afternoon in a fasted state, two hours before breaking the fast. The training sessions were supervised by fitness coaches. The training sessions and friendly soccer games were carried out at the same time (Fig. 2).

#### Group 1: Traditional Training (TT)

We adopted the same content of the training program applied before the Ramadan period, with a reduction in training volume per session by 15 min. TT was performed over four weeks with two weekly sessions, each lasting 60 min. Training contents of TT primarily comprised endurance and sprint-based exercises with fartlek, and high-intensity interval runs with the goal to enhance physical fitness.

#### Group 2: Neuromuscular Training (NMT)

NMT was conducted over four weeks with two weekly sessions. NMT included a warm-up, lower body strengthening, plyometrics, speed/agility, and core-stability exercises using bosu balls, mini-bands, and medicine balls (Table 3). Each session lasted 60 min and the training durations were similar between the TT and NMT groups.

**Table 3:** The four-week neuromuscular training (NMT) program applied in the study

Session	Week 1	Week 2	Week 3	Week 4	Exercises
Warm-up (7–10 min)	2 × 8 m	2 × 8 m	2 × 8 m	1 × 8 m	
Activation: 5 min (mini-band)					
Knee raises					
Butt kicks					
Open/Close hip					
Side step (Right/Left)					
Backward running					
Strengthening (7–15 min)					
Semi-squat (empty bar with elastic resistance)	2 × 15	3 × 15	3 × 15	4 × 15	
Forward lunge 10 m	2	3	3	4	
Hip thrust (with unstable surfaces 0.)	2 × 15	3 × 15	3 × 15	4 × 15	
Diver (with kettlebell 8 kg)	2 × 6	3 × 6	3 × 6	4 × 6	
Nordic hamstring reverse (medicine ball 4 kg)	2 × 8	3 × 8	3 × 8	4 × 8	
Copenhagen exercise (with unstable surfaces)	2 × 10 s	3 × 10 s	3 × 10 s	4 × 10 s	
Plyometrics (7–15 min)					
Bilateral forward hurdle jumps (45 cm)	2 × 4	3 × 4	3 × 4	4 × 4	
Single leg lateral hurdle jump (35 cm)	2 × 6	3 × 6	3 × 6	4 × 6	
Bilateral forward hurdle jump (25 cm)	2 × 6	3 × 6	3 × 6	4 × 6	
Single leg lateral hurdle jump (25 cm)	2 × 6	3 × 6	3 × 6	4 × 6	
Bounding (10 m) + sprint (10 m)	2 × 6	2 × 6	2 × 6	4 × 6	
Agility/speed (7–15 min)					
Change-of-direction slalom (CoD 90°)	2	3	3	4	
Acceleration/deceleration CoD slalom	2	3	3	4	
Shuttle run (CoD 180°)	2	3	3	4	
Core stability (7–15 min)					
Proprioception on Bosu ball	2 × 20 s	3 × 20 s	3 × 20 s	4 × 20 s	
Forward plank on Swiss ball					
Lateral plank /leg spread					
Crunches					
Lumbar extensions					

## Statistical Analyses

The normality of data distribution was assessed using the Shapiro–Wilk test. Data are presented as means and standard deviations (SD). A two-way ANOVA with the factors group (NMT, TT) and time (pre-test, post-test) was used to assess between and within group differences during the four training weeks. If a significant group-by-time interaction effect was detected, Bonferroni adjusted post-hoc tests (i.e., Student t-test) were computed. Effect sizes were estimated using Cohen's d (d) with demarcations of trivial (< 0.2), small (0.2–0.59), medium (0.60–1.19), large (1.2–1.99), and very large (≥ 2.0). To calculate Cohen’s *d*, we used eta squared (*η*^2^) from ANOVA for main time and group effects and group-by-time interactions. Percentage changes from pre-test to post-test were also calculated according to the following equation: (post-test values—pre-test values)/pre-test values × 100. The results were analyzed at a significance level of *p* < 0.05. All analyses were performed using SPSS for Windows IBM SPSS software (SPSS, version 28, Chicago; IL). Test–retest reliability of the variables was assessed using Cronbach’s model of ICCs and SEMs according to Peltola's method [35].

## Results

All players completed the study protocol as intended. There were no dropouts of the enrolled study participants. Pre-test (T1) values of all analyzed dependent variables were similar in both groups (Tables 1, 4).

**Table 4:** Effects of four weeks of training during Ramadan on anthropometric characteristics and body composition (means ± SDs)

Variables	Group	Pre	Post	Change %	Cohen’s d	ANOVA *p-value* (Cohen’s d)		
						Time	Group	Interaction
Body mass (kg)	TT	60.84 ± 5.26	62.01 ± 4.10	1.92	0.25	0.170 (0.25)	0.624 (0.27)	** < 0.001 (0.7)**
	NMT	61.95 ± 5.52	59.50 ± 3.63	− 3.95	0.52			
Body height (cm)	TT	171.23 ± 6.64	172.29 ± 6.69	0.62	0.15	** < 0.001 (1.04)**	0.527 (0.24)	0.223 (0.22)
	NMT	172.80 ± 6.76	173.39 ± 6.50	0.34	0.09			
Body fat (%)	TT	14.94 ± 2.52	14.30 ± 1.91	− 4.28	0.29	** < 0.001 (1.18)**	0.464 (0.44)	**0.002 (0.76)**
	NMT	15.25 ± 2.77	13.00 ± 1.63	− 14.75	0.99			
BMI (kg/m^2^)	TT	20.81 ± 2.17	20.99 ± 1.71	0.86	0.09	**0.042 (0.89)**	0.415 (0.77)	**0.004 (0.33)**
	NMT	20.85 ± 2.74	19.89 ± 1.67	− 4.60	0.42			

*TT* traditional training, *NMT* neuromuscular training, *BMI* body mass index, Bold indicates significant values

### Body Composition

Data on body composition are reported in Table 4. Significant group-by-time effects were observed for body mass (*p* < 0.001; d = 0.70), body fat (*p* = 0.002; d = 0.76), and BMI (*p* = 0.004; d = 0.33). Post-hoc tests indicated significant changes for body mass (*p* = 0.046; d = 0.65), body fat (*p* = 0.026; d = 0.73), and BMI (*p* = 0.047; d = 0.65) in the NMT but not in the TT group.

### Physical Fitness

Significant group-by-time effects were observed for linear sprint speed (10-m: *p* < 0.001, d = 2.58; 30-m: *p* = 0.049; d = 2.23), CoD speed (T-test without ball: *p* < 0.001, d = 1.03; T-test with ball: *p* < 0.001, d = 0.77), proxies of muscle power (SJ: *p* < 0.001, d = 4.04; CMJ: *p* < 0.001, d = 2.21), and soccer-related performance (YYIT L1: *p* = 0.005, d = 0.67; RSSA: *p* = 0.007, d = 0.46) (Table 5). Post-hoc tests revealed increases in physical fitness in the NMT but not the TT group for linear sprint speed (10-m: *p* < 0.001, d = 2.53; 30-m sprint: *p* < 0.001, d = 1.16), CoD speed (T-test: *p* < 0.001, d = 1.98; T-test with ball: *p* < 0.001, d = 2.36), proxies of muscle power (SJ: *p* < 0.001, d = 2.13; CMJ: *p* < 0.001, d = 1.51) and soccer-related performance (YYIT L1: *p* = 0.030, d = 0.71; RSSA: *p* < 0.001, d = 1.44).

**Table 5:** Effects of four weeks of training during Ramadan on physical fitness

Variables	Group	Pre	Post	Change %	Cohen’s d	ANOVA *p-value* (Cohen’s d)		
						Time	Group	Interaction
5-m Sprint (s)	TT	1.16 ± 0.06	1.19 ± 0.27	2.59	0.15	0.192 (4.86)	**0.027 (1.49)**	< 0.067 (2.16)
	NMT	1.15 ± 0.07	1.01 ± 0.23	− 12.17	0.82			
10-m Sprint (s)	TT	1.85 ± 0.09	1.92 ± 0.08	3.78	0.82	0.461 (5.27)	** < 0.001 (1.26)**	** < 0.001 (2.58)**
	NMT	1.82 ± 0.10	1.71 ± 0.07	− 6.04	1.27			
30-m Sprint (s)	TT	4.03 ± 0.10	4.04 ± 0.11	0.25	0.10	0.140 (4.28)	**0.011 (0.88)**	**0.049 (2.23)**
	NMT	4.00 ± 0.17	3.90 ± 0.11	− 2.50	0.70			
T-test (s)	TT	10.89 ± 0.47	10.98 ± 0.50	0.83	0.19	**0.002 (2.82)**	** < 0.001 (0.59)**	** < 0.001 (1.03)**
	NMT	10.96 ± 0.66	10.10 ± 0.37	− 7.85	1.61			
T-test with ball (s)	TT	13.72 ± 0.87	13.76 ± 0.56	0.29	0.06	** < 0.001 (2.33)**	** < 0.001 (0.34)**	** < 0.001 (0.77)**
	NMT	13.68 ± 0.86	12.53 ± 0.47	− 8.41	1.66			
SJ (cm)	TT	37.74 ± 3.00	35.85 ± 2.81	− 5.01	0.65	**0.038 (9.93)**	** < 0.001 (0.62)**	** < 0.001 (4.04)**
	NMT	37.67 ± 3.13	41.93 ± 2.91	11.31	1.41			
CMJ (cm)	TT	39.57 ± 2.95	38.23 ± 2.62	− 3.39	0.48	**0.036 (7.78)**	**0.016 (0.82)**	** < 0.001 (2.21)**
	NMT	39.17 ± 3.78	42.99 ± 3.61	9.75	1.03			
Yoyo L1 (m)	TT	2436 ± 365	2312 ± 343	− 5.09	0.35	0.451 (5.55)	0.167 (0.29)	**0.005 (0.67)**
	NMT	2494 ± 383	2568 ± 376	2.97	0.20			
RSSA decrement (%)	TT	6.28 ± 2.52	6.75 ± 1.61	7.48	0.23	0.075 (1.66)	**0.006 (0.06)**	**0.007 (0.46)**
	NMT	6.41 ± 1.56	4.29 ± 1.80	− 33.07	1.26			
LSPT TT (s)	TT	52.90 ± 6.21	54.20 ± 3.69	2.46	0.25	< 0.711 (5.78)	0.186 (0)	0.407 (1.08)
	NMT	52.45 ± 4.37	51.08 ± 4.00	− 2.61	0.33			

*TT* traditional training, *NMT* neuromuscular training, *SJ* squat jump, *CMJ* countermovement jump, *Yoyo L1* yoyo intermittent recovery test level 1, *RSSA* repeated-shuttle-sprint ability, *LSPT-TT* Loughborough soccer passing test total performance time; Values = means ± SDs; Bold indicates significant values

#### Isokinetic Strength Tests

Results for the isokinetic strength tests are displayed in Table 6. Significant group-by-time interactions were observed for knee extensor peak torque of the dominant limb (DL) Conc/Conc (60°/s) (*p* < 0.001, d = 2.21), knee extensor peak torque of the non-dominant limb (NDL) Conc/Conc (60°/s) (*p* < 0.001, d = 1.49), knee flexor peak torque of DL Conc/Conc (60°/s) (*p* = 0.001, d = 2.14), knee flexor peak torque of NDL Conc/Conc (60°/s) (*p* < 0.001, d = 1.33), knee extensor peak torque of DL Conc/Conc (240°/s) (*p* < 0.001, d = 1.12), knee extensor peak torque of NDL Conc/Conc (240°/s) (*p* < 0.001, d = 2.33), knee flexor peak torque of DL Conc/Conc (240°/s) (*p* < 0.001, d = 1.78), knee flexor peak torque of NDL Conc/Conc (240°/s) (*p* < 0.001, d = 2.65), knee extensor peak torque of DL Ecc /Ecc (30°/s) (*p* = 0.002, d = 1.04), knee extensor peak torque of NDL Ecc/Ecc (30°/s) (*p* = 0.001, d = 2.66), knee flexor peak torque of DL Ecc/Ecc (30°/s) (*p* = 0.001, d = 2.87), knee flexor peak torque of NDL Ecc/Ecc (30°/s) (*p* < 0.001, d = 4.22). Post-hoc tests revealed significant isokinetic strength increases in NMT compared to TT for knee extensor peak torque of DL Conc/Conc (60°/s) (*p* = 0.048, d = 0.65), knee extensor peak torque of NDL Conc/Conc (60°/s) (*p* = 0.031, d = 0.71), knee flexor peak torque of DL Conc/Conc (60°/s) (*p* = 0.044, d = 0.66), knee flexor peak torque of NDL Conc/Conc (60°/s) (*p* = 0.046, d = 0.65), knee extensor peak torque of DL Conc/Conc (240°/s) (*p* = 0.049, d = 0.64), knee extensor peak torque of NDL Conc/Conc (240°/s) (*p* = 0.049, d = 0.64), knee flexor peak torque of DL Conc/Conc (240°/s) (*p* = 0.015, d = 0.81), knee flexor peak torque of NDL Conc/Conc (240°/s) (*p* = 0.029, d = 0.72), knee extensor peak torque of DL Ecc/Ecc (30°/s) (*p* = 0.046, d = 0.65), knee extensor peak torque of NDL Ecc/Ecc (30°/s) (*p* = 0.044, d = 0.66), knee flexor peak torque of DL Ecc/Ecc (30°/s) (*p* = 0.039, d = 0.68), knee flexor peak torque of NDL Ecc/Ecc (30°/s) (*p* = 0.042, d = 0.67).

**Table 6:** Effects of four weeks of training during Ramadan on isokinetic muscular strength tests

Variables	Group	Pre	Post	Change %	Cohen’s d	ANOVA *p-value* (Cohen’s d)		
						Time	Group	Interaction
Extensors 60°/s conc PT (Nm)								
Dominant limb	TT	158.33 ± 18.01	158.18 ± 11.60	− 0.09	0.01	** < 0.001 (2.28)**	0.524 (0.71)	** < 0.001 (2.21)**
	NMT	156.47 ± 14.75	165.69 ± 11.68	5.89	0.69			
Non-dominant limb	TT	143.30 ± 14.01	142.23 ± 15.10	− 0.75	0.07	**0.005 (**2.08**)**	0.145 (0.68)	** < 0.001 (1.49)**
	NMT	145.95 ± 13.05	152.45 ± 13.65	4.45	0.49			
Flexors 60°/s conc PT (Nm)								
Dominant limb	TT	123.51 ± 17.66	122.24 ± 12.92	− 1.03	0.08	**0.050 (1.88)**	0.234 (0.86)	**0.001 (2.14)**
	NMT	125.49 ± 9.88	130.16 ± 11.07	3.72	0.45			
Non-dominant limb	TT	110.49 ± 16.11	110.48 ± 13.03	− 0.01	0.00	** < 0.001 (2.76)**	0.257 (0.74)	** < 0.001 (1.33)**
	NMT	112.15 ± 12.47	118.37 ± 11.12	5.55	0.53			
Extensors 240°/s conc PT (Nm)								
Dominant limb	TT	124.68 ± 15.17	123.13 ± 11.43	− 1.24	0.12	**0.003 (1.03)**	0.522 (0.56)	** < 0.001 (1.12)**
	NMT	122.88 ± 14.17	130.06 ± 10.08	5.84	0.58			
Non-dominant limb	TT	112.43 ± 11.68	111.69 ± 9.45	− 0.66	0.07	** < 0.001 (1.29)**	0.678 (0.04)	** < 0.001 (2.33)**
	NMT	109.32 ± 12.59	117.55 ± 8.79	7.53	0.76			
Flexors 240°/s conc PT (Nm)								
Dominant limb	TT	111.51 ± 9.71	109.50 ± 9.30	− 1.80	0.21	**0.016 (1.16)**	0.271 (0.32)	** < 0.001 (1.78)**
	NMT	111.05 ± 13.38	117.32 ± 10.08	5.65	0.53			
Non-dominant limb	TT	103.47 ± 10.04	101.43 ± 9.47	− 1.97	0.21	**0.011 (1.26)**	0.392 (0.59)	** < 0.001 (2.65)**
	NMT	101.97 ± 11.56	108.37 ± 9.82	6.28	0.60			
Extensors 30°/s ecc PT (Nm)								
Dominant limb	TT	138.88 ± 16.42	138.26 ± 12.45	− 0.45	0.04	**0.012 (1.05)**	0.293 (0.50)	**0.002 (1.04)**
	NMT	140.72 ± 17.91	146.31 ± 12.28	3.97	0.36			
Non-dominant limb	TT	127.35 ± 13.99	126.89 ± 11.94	− 0.36	0.04	**0.004 (1.88)**	0.229 (0.65)	**0.001 (2.66)**
	NMT	129.62 ± 15.90	135.10 ± 12.99	4.23	0.38			
Flexors 30°/s ecc PT (Nm)								
Dominant limb	TT	166.99 ± 23.85	166.18 ± 14.20	− 0.49	0.04	**0.005 (1.55)**	0.492 (0.33)	**0.001 (2.87)**
	NMT	165.05 ± 19.05	175.80 ± 14.27	6.51	0.64			
Non-dominant limb	TT	152.77 ± 20.68	151.91 ± 13.93	− 0.56	0.05	** < 0.001 (2.65)**	0.610 (0.12)	** < 0.001 (4.22)**
	NMT	149.17 ± 18.77	160.80 ± 12.73	7,80	0.73			
Conventional ratio H conc 240°/s / Q conc 240°/s								
Dominant limb	TT	0.90 ± 0.09	0.89 ± 0.11	− 1.11	0.10	0.551 (0.24)	0.842 (0.11)	0.551 (0.18)
	NMT	0.90 ± 0.04	0.90 ± 0.04	0.00	0.00			
Non-dominant limb	TT	0.92 ± 0.09	0.91 ± 0.08	− 1.09	0.12	0.144 (0.88)	0.571 (0.22)	0.902 (0.32)
	NMT	0.93 ± 0.05	0.92 ± 0.07	− 1.08	0.16			
Functional ratio H ecc 30°/s / Q conc 240°/s								
Dominant limb	TT	1.34 ± 0.14	1.35 ± 0.09	0.75	0.08	0.724 (0.19)	0.919 (0.12)	0.797 (0.16)
	NMT	1.35 ± 0.15	1.35 ± 0.07	0.00	0.00			
Non-dominant limb	TT	1.36 ± 0.16	1.36 ± 0.12	0.00	0.00	0.921 (0.16)	0.839 (0.23)	0.832 (0.17)
	NMT	1.38 ± 0.21	1.37 ± 0.12	− 0.72	0.06			

*TT* traditional training; *NMT* neuromuscular training; *PT* peak torque; *BMI* body mass index; *conc*. concentrique; *ecc*. eccentric; *H* hamstring; *Q* quadriceps; *DF* dominant foot; *NDF* non-dominant foot; Values = mean ± SD; Bold indicates significant values

### Hooper Questionnaire

Hooper questionnaire scores obtained during Ramadan did not show any significant differences between the TT (11.89 ± 2.60 AU) and NMT groups (11.96 ± 2.38 AU) during the study (*p* = 0.967; d = 0.03). Furthermore, no significant differences were observed between TT and NMT for sleep quality (3.54 ± 0.58, 3.57 ± 0.51; *p* = 0.931; d = 0.05), stress (2.64 ± 0.78, 2.54 ± 0.96; *p* = 0.870; d = 0.11), delayed onset muscle soreness (DOMS, 2.79 ± 0.63, 2.89 ± 0.70; *p* = 0.832; d = 0.15), and fatigue (2.93 ± 0.74, 2.96 ± 0.55; *p* = 0.938; d = 0.05), respectively (Table 7).

**Table 7:** Hooper index (sleep, stress, DOMS, and fatigue) data according to the training program, (means ± SDs)

	Group	Sleep (AU)	Stress (AU)	DOMS (AU)	Fatigue (AU)	Hooper’s index (AU)
Week 1	TT	2.71 ± 0.76	1.57 ± 0.53	1.86 ± 1.07	2.00 ± 0.82	8.14 ± 1.68
	NMT	3.14 ± 1.07	1.29 ± 0.49	1.86 ± 0.69	2.14 ± 1.07	8.43 ± 2.57
Week 2	TT	3.57 ± 0.98	2.86 ± 0.69	3.00 ± 0.58	2.71 ± 0.95	12.14 ± 2.04
	NMT	3.86 ± 0.90	2.43 ± 0.79	3.14 ± 0.90	3.29 ± 1.25	12.71 ± 2.29
Week 3	TT	4.00 ± 1.15	2.71 ± 1.25	3.29 ± 1.11	3.71 ± 1.11	13.71 ± 3.15
	NMT	4.14 ± 1.07	2.86 ± 1.21	3.14 ± 1.07	3.29 ± 0.49	13.43 ± 2.30
Week 4	TT	3.86 ± 0.69	3.43 ± 0.98	3.00 ± 1.29	3.29 ± 0.49	13.57 ± 3.10
	NMT	3.14 ± 0.90	3.57 ± 0.79	3.43 ± 0.79	3.14 ± 1.57	13.29 ± 2.98
Means	TT	3.54 ± 0.58	2.64 ± 0.78	2.79 ± 0.63	2.93 ± 0.74	11.89 ± 2.60
	MMT	3.57 ± 0.51	2.54 ± 0.96	2.89 ± 0.70	2.96 ± 0.55	11.96 ± 2.38
p-value (Cohen's d)		0.93 (0.05)	0.87 (0.11)	0.83 (0.15)	0.93 (0.05)	0.96 (0.03)

*DOMS* delayed-onset muscle soreness; *TT* traditional training; *NMT* neuromuscular training; *AU* arbitrary unit

### Training Load

No significant difference was observed regarding the internal load (sRPE) between TT (1444 ± 77 AU) and NMT (1474 ± 65 AU) during the study (*p* = 0.287; d = 0.42). The external load, which was measured using GPS data, indicated no significant difference in total distance, moderate intensity distance (15–20 km/h) and high-intensity distance (20–25 km/h). However, the GPS data indicated significant differences in favor of the NMT group for sprint distances > 25 km/h (*p* = 0.046; d = 1.42), number of sprints (*p* = 0.003; d = 2.89), accelerations (*p* = 0.002; d = 3.20), and decelerations (*p* = 0.001; d = 3.49) (Table 8).

**Table 8:** Indicators of external (GPS data) and internal load (RPE) of players during the Ramadan period (means ± SDs).

	Group	Indicators of external load (GPS data)							Indicators of internal load (session RPE)
		TD	15–20 km	20–25 km	> 25 km	Sprint event	Acc event	Dec event	A.U
Week 1	TT	4241 ± 2437	1903 ± 853	91 ± 76	55 ± 40	24 ± 13	16 ± 8	16 ± 9	1335 ± 130
	NMT	4089 ± 2233	1868 ± 881	93 ± 72	69 ± 50	32 ± 19	19 ± 11	20 ± 10	1410 ± 136
Week 2	TT	4502 ± 2753	2149 ± 1156	93 ± 59	60 ± 54	19 ± 9	16 ± 9	15 ± 9	1470 ± 136
	NMT	4369 ± 2564	1812 ± 1008	93 ± 62	63 ± 45	27 ± 17	25 ± 14	28 ± 14	1560 ± 156
Week 3	TT	4675 ± 2542	1391 ± 845	75 ± 62	67 ± 44	19 ± 10	17 ± 8	16 ± 8	1515 ± 128
	NMT	4384 ± 2236	1621 ± 837	105 ± 74	80 ± 53	26 ± 15	26 ± 14	28 ± 16	1440 ± 130
Week 4	TT	5237 ± 2615	1940 ± 1012	88 ± 57	68 ± 44	17 ± 8	15 ± 8	17 ± 9	1455 ± 147
	NMT	5000 ± 2603	1955 ± 990	91 ± 53	79 ± 53	27 ± 15	23 ± 12	27 ± 14	1485 ± 152
Means	TT	4664 ± 422	1796 ± 326	87 ± 8	62 ± 6	20 ± 3	16 ± 1	16 ± 1	1444 ± 77
	NMT	4460 ± 384	1814 ± 142	96 ± 6	73 ± 8	28 ± 3	23 ± 3	26 ± 4	1474 ± 65
*p*-value (Cohen’s d)	0.25 (0.50)	0.46 (0.07)	0.07 (1.20)	**0.04 (1.42)**	**0.003 (2.89)**	**0.002 (3.20)**	**0.001 (3.49)**	0.28 (0.42)	

*TT* traditional training; *NMT* neuromuscular training; *RPE* ratings of perceived exertion; *GPS* global positioning system; *TD* total distance; *Acc event* acceleration event; *Dec event* deceleration event; *AU* arbitrary unit; Bold indicates significant values

### Injury Occurrence

**Fig. 3:**
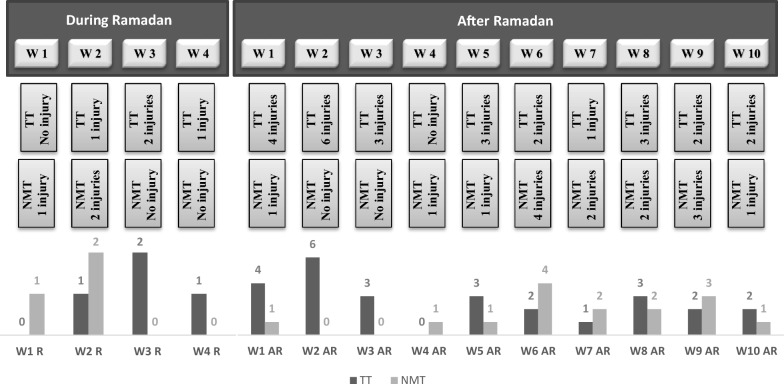
Occurrence of injuries per week during and after Ramadan. *W* week; *R* during Ramadan; *AR* after Ramadan; *TT* traditional training; *NMT* neuromuscular training.

The injury data recorded during and after Ramadan periods are presented in Fig. 3 and Table 9.

**Table 9:** Location, mechanisms, types, severity, burden and incidence of injury during and after Ramadan.

	During Ramadan			After Ramadan		
	TT	NMT	Total	TT	NMT	Total
Injury location (% of total)						
Upper limbs	–	–	–	1 (4%)	2 (13%)	3 (6%)
Trunk	–	–	–	1 (4%)	2 (13%)	3 (8%)
Lower limbs	4 (100%)	3 (100%)	7 (100%)	24 (92%)	11 (74%)	35 (86%)
Injury mechanisms (% of total)						
Contact	2 (50%)	2 (67%)	4 (57%)	11 (42%)	13 (87%)	24 (59%)
Non-contact	2 (50%)	1 (33%)	3 (43%)	15 (58%)	2 (13%)	17 (41%)
Injury type (% of total)						
Fracture and bone stress	–	–	–	–	–	–
Joint (non bony structure) and ligament	1 (25%)	1 (33%)	2 (29%)	3 (11%)	1 (7%)	4 (10%)
Muscle and tendon	1 (25%)	–	1 (14%)	11 (42%)	1 (7%)	12 (29%)
Contusions	2 (50%)	2 (67%)	4 (57%)	11 (43%)	13 (86%)	24 (59%)
Laceration and skin lesion	–	–	–	1 (4%)	–	1 (2%)
Severity (% of total)						
Minimal (1–3 days)	4	3	7	15 (58%)	9 (60%)	24 (59%)
Minor (4–7 days)	–	–	–	7 (27%)	5 (33%)	12 (29%)
Moderate (8–28 days)	–	–	–	3 (12%)	1 (7%)	4 (10%)
Severe (+ 29 days)	–	–	–	1 (3%)	0 (0%)	1 (2%)
Total injuries	4	3	7	26	15	41
Burden						
Total days lost	12	8	20	157	62	219
Average days lost per player	0.60	0.40	0.50	7.85	3.10	5.48
Overall exposure time (a)	500	500	1000	1750	1750	3500
Injury burden (b)	24.00	16.00	20.00	89.71	35.42	62.57
Incidence injury rate (IRR)						
IIR Training						
Number of training injuries (c)	2	1	3	12	5	17
Exposure time training	440	440	880	1600	1600	3200
IIR (d)	4.55	2.27	3.41	7.50	3.13	5.31
IRR (95% CI) (e)	0.50 [0.27–0.93]			0.42 [0.22–0.78]		
IIR Match						
Number of match injuries	2	2	4	14	10	24
Exposure time match	60	60	120	150	150	300
IIR	33.33	33.33	33.33	93.33	66.67	80.00
IRR (95% CI)	1.00 [0.54–1.86]			0.71 [0.38–1.33]		
IIR Overall	8.00	6.00	7.00	14.86	8.57	11.71
IRR (95% CI)	**0.75 [0.40–1.39]**			**0.58 [0.31–1.07]**		

*TT* traditional training; *NMT* neuromuscular training. (a) Overall exposure time: exposure training time calculated using number of players per training session (around 40 players per group). Number of training session (around 20 for the Ramadan period and 70 for the after Ramadan period) duration of session per min (45–75 min for the Ramadan period and 60–120 min for the after Ramadan period). Exposure match time was calculated using of number of played matches (10 matches for the studied period). Number of players during the game (10 players) and duration of match per min (90 min). (b) burden = 1000 × (∑ days absent/ ∑ exposure hours). (c) Number of injuries was recorded during training and matches. (d) IIR (incidence injury rate) was calculated, IIR = 1000 × (∑ injuries/ ∑ exposure hours) (Fuller CW. et al. 2006). (e) *IRR* incidence rate ratio

#### During Ramadan Fasting

During Ramadan, seven injuries were recorded (Fig. 3), including four contusions, two sprains and one muscle injury. The most frequently occurring injury types were contusions, with two injuries in the TT group (50%) and the NMT group (67%), followed by joint and ligament injuries with one injury in the TT group (25%) and the NMT group (33%), muscle and tendon injuries, and one injury in the TT group. All injuries were recorded for the lower limb whether in the TT or NMT group. The mechanism of injury was also recorded. Inthe TT group, half of the injuries were contact injuries (two injuries, 50%) and the other half were non-contact injuries (two injuries, 50%), in the NMT group two (67%) of the injuries recorded were with contact and one (33%) injury was non-contact (Table 9).

The time of absence and the severity of injuries were also recorded. In the TT group, 12 days of absence (average days lost per player = 0.60) were recorded with minimal severity (1–3 days). In the NMT group, eight days of absence (average days lost per player = 0.40) were recorded with minimal severity (1–3 days). Regarding the injury burden, there were 24 days absent/1000 h for the TT group, and 16 days /1000 h for the NMTgroup (Table 9).

The injury incidence recorded during the training period of Ramadan was 4.55 injuries/1000 h in the TT group and 2.27 injuries/1000 h in the NMT group and an incidence rate ratio of 0.50 (95% CI 0.27–0.93). However, the injury incidence recorded during the match period was identical in both groups with 33.33 injuries/1000 h and an incidence rate ratio of 1.00 (95% CI 0.54–1.86). The overall injury incidence recorded was 8.00 and 6.00 injuries/1000 h in the TT and NMT group, respectively and an incidence rate ratio of 0.75 (95% CI 0.40–1.39) (Table 9).

#### After Ramadan Fasting

After the Ramadan period, 41 injuries were recorded (Fig. 3) with 26 in the TT group and 15 in the NMT group. The most frequent injury types were contusions with eleven injuries (43%) in the TT group and 13 injuries (86%) in the NMT group, then muscle and tendon injuries, eleven injuries (42%) in the TT group and one injury (7%) in the NMT group, ligament injuries, three injuries (11%) in the TT group and one injury (7%) in the NMT group, lacerations, one injury (4%) in the TT group (Table 9). In both experimental groups, the most injuries were recorded in the lower limbs (Table 9). The mechanism of injury was also moniored. In the TT group, eleven (42%) of the injuries recorded were with contact and 15 (58%) injuries were non-contact. In the NMT group, 13 (79%) of the injuries reported were with contact and two (13%) injuries were non-contact (Table 9).

Regarding the time of absence and the severity of injuries, in the TT group, 157 days of absence (average days lost per player = 7.85) were recorded, including 15 minimal injuries (1–3 days), seven minor injuries (4–7 days), three moderate injuries (8–28 days), one severe injury (+ 29 days). In the NMT group, 62 days of absence (average days lost per player = 3.10) were recorded including nine minimal injuries (1–3 days), five minor injuries (4–7 days), one moderate injury (8–28 days), no severe injury (+ 29 days) (Table 9). The injury burden was also calculated. In the TT group, 89.71 days absent/1000 h and 35.42 days absent/1000 h. (Table 9).

In this study, the injury incidence recorded during the training period after Ramadan was 7.50 injuries/1000 h in the TT group and 3.12 injuries/1000 h in the NMT group and an incidence rate ratio of 0.42 (95% CI 0.22–0.78). However, the injury incidence recorded during the match period was 93.33 injuries/1000 h in the TT group and 66.67 injuries/1000 h in the NMT group and an incidence rate ratio of 0.71 (95% CI 0.38–1.33). The overall injury incidence recorded was 14.86 injuries/1000 h and 8.57 injuries/1000 h in the TT and NMT group respectively and an incidence rate ratio of 0.58 (95% CI 0.31–1.07) (Table 9).

The overall injury rate during and after Ramadan fasting period was significantly lower in NMT (8.00/1000 h exposure) compared to TT (13.33/1000 h exposure) (*p* = 0.049; d = 0.66).

## Discussion

### Body Composition

Adequate body composition in the form of low body fat and high lean mass contribute to athletes' success in soccer, in addition to physical, physiological, genetic and psychological factors [36]. Furthermore, low fat mass and high lean mass are also linked to better performances as observed by Alkandari et al. [37] in young and adult athletes participating in vigorous sports (> 2400 Met.min/week). During the intermittent fasting period of Ramadan, youth and adult soccer players continue to train and compete, no matter whether training sessions take place before or after breaking the fast.

The results of our study demonstrate significant decreases in body mass, percentage of fat mass and body mass index in the NMT group. According to Trabelsi et al. [38], physical activity during fasting can increase the oxidation of fatty acids as energy substrates, leading to significant losses of body fat [38]. Furthermore, decreases in body mass during the Ramadan period may be associated with the increased use of body fat stores as an energy source at rest and during physical exercise [39]. The reduction in body mass reported during Ramadan in some studies was attributed to the increased use of body fat stores as an energy substrate, both at rest and during exercise [38, 39].

Our findings agree with other studies reporting that strength training associated with intermittent fasting reduced fat mass and improved body composition [40, 41]. Studies conducted outside of Ramadan also observed that NMT induced a significant decrease in body mass and % fat mass in soccer players [42]. However, other studies did not observe changes in body and fat mass [43]. Furthermore, Trabelsi et al. [44] reported that training at least three times a week during the Ramadan period had no effect on body mass and body composition in adolescent athletes, likely due to similar total energy and water intake during Ramadan compared to before Ramadan [44].

### Physical Fitness

#### Proxies of Muscle Power

Our results demonstrate improvements in SJ (+ 11.3%) and CMJ (+ 9.7%) for the NMT group without changes in the TT group, which is in agreement with other studies demonstrating that NMT associated with intermittent fasting improved muscular fitness and also jumping performance [19, 20]. Indeed, researchers analyzing the combined effect of strength training and intermittent fasting reported improvements in muscle strength, muscle power, linear sprint and CoD speed in young players [18, 45]. This study indicates that intermittent fasting combined with a four-week NMT improved performance levels in NMT compared to TT. The observed substantial improvements in muscle strength and power are multi-factorial. First, physiological adaptations like increased muscle mass and changes in body composition, may have contributed to the observed improvements in force production capacity [46, 47]. Second, neural adaptations induced by NMT [48] such as improved motor unit recruitment and firing frequency may have enhanced muscle activity leading to greater force output. Finally, NMT exercises such as the hip-thrust, half-squat, forward lunge, plyometrics and core stability exercises [49–53], could have played a central role in improving muscle strength and power. These exercises aim at inducing structural (muscle size) and neural adaptations (motor unit recruitment, firing frequency), essential to maximize performance [49–53].

#### Linear Sprint and Change-of-Direction Speed

Our study demonstrated significant group-by-time interaction effects for linear sprint (10 and 30-m) and CoD speed in the NMT group, resulting in improved performance times in the 10 and 30-m sprints (− 6% and − 2.5%), T-test with ball (− 8.41%) and without ball (− 7.85%), and significant main effects in the 5-m sprint (− 12%). Our findings are in agreement with other studies on improvements of speed and CoD performances due to NMT in young soccer players [45, 54]. Indeed, most studies that showed an improvement in CoD speed and/or agility incorporated NMT exercises aimed at improving CoD speed and/or agility [55, 56]. In addition, the observed changes in body composition could promote, at least in part, gains in speed as well CoD speed [57]. Moreover, some researchers reported that Ramadan intermittent fasting does not affect linear sprint and CoD speed performance in young soccer players [58, 59]. Indeed, performance of short-term efforts was well maintained during the Ramadan period, likely because short completion time exposes players to lower levels of fatigue [58, 60].

#### Soccer-Specific Performance Tests

##### Yo–Yo Intermittent Recovery Level 1 (YYIRT L1) and Repeated Shuttle Sprint Ability (RSSA)

Our study indicates improvements in YYIRTL1 and RSSA tests in the NMT group compared to the TT group. Some researchers reported that NMT should be considered as a complementary method to aerobic conditioning in youth soccer players, given the multidirectional nature of the game and the need to cover long distances [54]. In addition, improvements in physical fitness during the Ramadan intermittent fasting period can also be explained by the physiological adaptation of the athlete following NMT [1, 61].

##### The Loughborough Soccer Passing Test (LSPT)

Our study indicates non-significant changes in LSPT performance in the NMT group. The results of our study are in agreement with the data of [62] who reported that four weeks of NMT in young soccer players induced small improvements in the LSPT test. The LSPT can assess both soccer-specific skills and cognitive function in young soccer players, and the absence of larger improvement in our study may be due to a persistent alteration in the players' post-Ramadan cognitive function and by the short duration of the experimental program (four weeks). Indeed, most researchers reporting positive effects of NMT on the technical performance of young players had longer durations (e.g., at least 12 weeks) compared to our study [61, 63]. Other researchers did not observe negative effects of Ramadan intermittent fasting on soccer-specific performances in young players [58, 59]. For example, Kirkendall et al. [59] indicated that Ramadan intermittent fasting had an effect on LSPT performance in young soccer players.

However, it is important to point out that many researchers have reported a negative impact of intermittent fasting on physical performance in young soccer players [64–69]. The discrepancy in findings between previous research and ours can amongst others be explained by the training programs adopted during Ramadan fasting. Previously, researchers have applied traditional training programs and reduced the training loads [64–69]. Findings from our study are novel since we applied NMT as training program.

### Isokinetic Strength Tests

Maintaining the joint stability of the players' knee is ensured by the muscular balance of the thigh. Thus, we measured the muscular strength of the extensors (quadriceps) and flexors (hamstrings) of the knee using an isokinetic dynamometer [25]. In addition, we calculated the ratios (Hcon / Qcon and Hecc / Qcon) in order to measure the imbalances of the agonist and antagonist muscles and analyze the potential risks of injuries in young players [27]. Indeed, it has been reported that players who have low ratios are more exposed to injuries [27]. Our results demonstrate an improvement of the knee extensor peak torque and the knee flexor peak torque in the three angular velocities that we used (Con 60°/s, Con 240°/s and Ecc 30°/s) and in both limbs (dominant and non-dominant). We can explain this improvement by the beneficial effect of NMT which allows an improvement in the coordination between the nervous system and the muscles, which translates in muscular strength into better recruitment and better synchronization of the motors units requested [70, 71]. In addition, it is important to specify that this improvement in the extensor and flexor peak torque could allow players to better perform power-related muscle actions specific to soccer (jumping, sprinting and shooting) [72] and to reduce the risk of injuries by improving the antagonistic activity of the hamstrings which is essential for the knee deceleration, thereby controlling the movement of the joint [73, 74]. However, our results did not show a significant group-by-time effect of the ratios calculated for both training groups. Our results can be explained by the duration of the short program (four weeks) which would have hindered the significant improvement in the ratios. Therefore, we assume that the lack of significant improvement after NMT requires additional training stimuli in the form of longer training duration to improve performance. Nevertheless, our ratios indicate a higher value than the thresholds defined in the literature [26, 27]. That is, it has been shown that a conventional ratio below 0.60 and a functional ratio below 1.0 can expose players to hamstring and anterior cruciate ligament injuries [27].

### Injuries

#### Location of Injuries

All recorded injuries during the intermittent Ramadan fasting period were located at the lower limb in both groups (NMT and TT). In addition, a high percentage of the injuries that we recorded during the post-Ramadan period were located in the lower limb (NMT, 74%; TT, 92%) which is similar to other reports in the literature [75, 76]. This can most likely be explained by the nature of soccer activity which mainly involves the lower limb in different training and match play actions such as running, sprinting, jumping, accelerating, decelerating and changing directions mainly putting load on the lower limbs [77].

#### Injury Mechanism and Type of Injuries

The results of our study showed a low prevalence of non-contact injuries in the NMT group compared to the TT group, whether during Ramadan 43% and 50% respectively or after Ramadan 13% and 58%. Our data are in agreement with the data from the systematic review and meta-analysis of Robles-Palazón et al. [76] who showed that NMT allows the reduction and prevention of non-contact injuries in young soccer players [76, 78]. After the Ramadan period, muscle–tendon injuries followed by joint/ligament (non-osseous) injuries represented the most common type of non-contact injuries in the TT group with approximately 34% of muscle–tendon injuries recorded all at the thigh including: five contractures, three strains, one tear and 8% tendon injuries. In the NMT group, we recorded a single muscle injury to the adductors (one strain). Regarding joint/ligament (non-osseous) injuries, our study recorded 11% of injuries in the TT group and 7% in the NMT group of sprain type.

Our results can be explained by the preventive impact of NMT which has likely reduced the risk factors for muscle/tendon and joint/ligament injuries in the NMT group. Our results are in agreement with the conclusions of the systematic review and meta-analysis of Robles-Palazón et al. [76] indicating that this type of non-contact injuries is the most common among young male soccer players [76]. Additionally, these results support similar findings observed in previous studies [76, 77] reporting that muscle–tendon injuries in the thigh and joint/ligament injuries in the ankle were the most common injuries among young players [79, 80]. Contusions represented the most common type of overall injury after Ramadan in both training groups, with approximately 86% in the NMT group and 43% in the TT group. This can be explained by the increase in match play intensity and impact during the post-Ramadan period. Indeed, the post-Ramadan period coincided with the play-off competition period.

#### Severity of Injuries

The impact of injuries was measured based on the duration of absence from regular team training and matches. Injuries of minimal severity (1–3 days), minor severity (4–7 days), moderate severity (8–28 days) and severe injuries (> 28 days) were recorded [34]. The data from our study showed that there were only minimal injuries (absence 1–3 days) during the Ramadan period and a majority of minimal injuries after Ramadan in both groups with approximately 58% in the TT group and 60% in the NMT group.

Our results are in agreement with the data from authors who reported that the prevalence of injuries among young soccer players is of minimal severity [76]. Furthermore, our data shows that the NMT group recorded a lower average number of days of absence per player (3.10 days of absence per player) compared to the TT group (7.85 days of absence per player) with an overall injury burden of 89.71 days absent/1000 h for TT group and 35.42 days absent/1000 h for NMT. Our findings align with the data from other researchers, who demonstrated that NMT could lead to a reduction in the number of days of absence and thus in the burden injury of players during training and matches [76, 81].

#### Incidence Rate of Injuries

In order to compare the effects of NMT on injury prevention, we calculated the overall injury incidence rate (IIR), during training and matches and during and after the Ramadan period.

Our results reported a lower IIR during training in Ramadan for the NMT group (4.55 per 1000 h of exposure) compared to the TT group (2.27 per 1000 h of exposure) and an IIR ratio 0.50 (95% CI 0.27–0.93). The IIR during matches in Ramadan (33.33 per 1000 h of exposure) did not show any difference between the two groups. However, the overall IIR during the intermittent fasting period was lower for the NMT group (6.00 per 1000 h of exposure) compared to the TT group (8.00 per 1000 h of exposure) with an IIR Ratio approximately of 0.75 (95% CI 0.40–1.39). Our data demonstrate the effects of NMT on injury prevention during the Ramadan period. Our results confirm the data of Cay et al. [82] who demonstrated that NMT including balance exercises helps prevent the occurrence of injuries during the Ramadan period [82].

During the period following the observance of Ramadan until the end of the season, our results indicated a lower overall IIR for the NMT group (8.57 per 1000 h of exposure) compared to the TT group (14.86 for 1000 h of exposure). In addition, we observed a higher IIR during matches compared to training for both groups of approximately 93.33 per 1000 h matches for the TT group and 66.67 for the NMT group. However, for the IIR during training, our data indicated approximately 7.50. for the TT group and 3.13 for 1000 h of exposure for the NMT group. These results confirm the positive effects of NMT in reducing the IIR during matches and training as reported by several authors [76, 78]. Furthermore, our results show an IIR ratio during training of 0.42 (95% CI 0.22–0.78), an IIR ratio during matches of 0.71 (95% CI 0.38–1.33) and an overall IIR ratio of 0.58 (95% CI 0.31–1.07), indicating lower injury rates during the post-Ramadan period in the NMT group compared to the TT group.

## Study Limitations

Our study has some limitations that need to be considered: (i) our results cannot be applied to all soccer players because the study was carried out with a sample of players from the same soccer academy and further research is needed to verify these results with other samples (e.g., different expertise level, sex); (ii) our study included only youth male players, making it difficult to apply these findings to female players or older male players; (iii) we did not measure changes in sleep quantity and its impact on changes in cognitive function; (iv) we did not control the outside-of-training physical activity that the players may have conducted or their food consumption outside of the scheduled meals. Moreover, even though the players were encouraged to eat and completely finish their dishes, dietary intakes were not objectively monitored; and finally, (v) a key limitation of our study is the short duration of the intervention, which was restricted to four weeks (Ramadan period). Longer training periods might yield different outcomes or reveal more pronounced effects.

## Practical Implications

- The integration of 60 min NMT during Ramadan, including strengthening, jumping, linear sprint and CoD speed, core stability and balance exercises, two times per week, for four-weeks improved body composition, physical fitness, and soccer-specific performance in young male and highly-trained soccer players.
- The incorporation of NMT, two times per week during Ramadan fasting can reduce the risk of injury by improving isokinetic strength and ratios.
- The present study demonstrated that NMT has the potential to reduce the number of injuries during the Ramadan period by more than 57% and after Ramadan by more than 60% compared to TT.
- This study has implications for coaches, particularly with regards to the type of training (i.e., NMT) they can use during Ramadan to optimize the physical fitness of their players.
- The results on the effectiveness of NMT could potentially be transferred to other sports such as basketball, handball, rugby or even athletics, where physical fitness and injury prevention play a key role for athletes’ performance and health.
- NMT could be used to extend the careers of older athletes by lowering the risk of injury through performance improvements in balance, core strength and change-of-direction speed.

## Conclusions

In summary, this is likely the first study to examine the impact of NMT applied during Ramadan on body composition, physical fitness including isokinetic muscle strength, and injury occurrence in young male and highly-trained soccer players. Findings of this study confirm the initial study hypotheses in as much as we found positive effects of NMT when applied during the intermittent Ramadan fasting period on physical fitness and injury prevention in young male and highly-trained soccer players. Future research could examine different exercise intensities and how they moderate the observed findings on physical fitness and injury prevention. Researchers could additionally explore the effects of NMT in a wider range of athletic populations, including females, athletes from different sports, and individuals at various competitive levels, to determine the generalizability of the findings.

## Data Availability

All data supporting the findings of this study are available upon a reasonable request to the corresponding authors.

## Abbreviations

ANOVA: Analysis of variance
CMJ: Countermovement jump
CoD: Change of direction
ET: Endurance-dominated training
GPS: Global positioning system
ICC: Intra-class correlation coefficients
CI: Confidence interval
LSPT: Loughborough soccer passing test
NMT: Neuromuscular training
RM: Repetition maximum
RSSA: Repeated shuttle sprint ability
Session-RPE: Session rating of perceived exertion
SJ: Squat jump
YYIRT 1: Yo–Yo intermittent recovery test 1
